# On Surgical Operations

**Published:** 1856-03

**Authors:** 


*On Surgical Operations.* By SENEX, of Virginia.

The department of surgery is universally admitted, by the enlightened members of tho medical profession, to be the most difficult branch of the whole round into which medicine has been distributed as a practical profession; whether contemplated in reference to the numerous and diversified diseases that fall properly within the province of the surgeon, or the many painful and dangerous operations demanded at his hands for the cure of those diseases. Hence, in all probability, is furnished a satisfactory reason why so few medical men, of the large number that study medicine as a profession, become operative surgeons. The duties of a practical surgeon are indeed onorous aud important, and well calculated to deter beginners, in the onset of their professional career, from attempts to operate in dangerous cases, and even from witnessing painful and bloody operations in some instances. With beginners thus constituted, nothing but a sense of duty or humanity could operate as inducements to attempt a critical and hazardous surgical operation; and in many instances humanity has had to lament the insufficiency of these powerful incentives, where life might have been saved by a timely surgical operation. So revolting and horrifying to the feelings are many of the operations, as to have caused veteran surgeons in some instances to pause and to waver before consenting to undertake them, or even to advise such operations.

It is highly probable that every humane and conscientious surgeon is the subject of such emotions, in greater or less degree, before commencing or even advising painful and dangerous operations; and, if it is a weakness to yield to such a struggle of feeling, the victory is due to the surgeon's humanity chiefly; and while it does not compromise his moral courage, is creditable to his heart as a man, and generally leads to the safety of the afflicted, as it does to virtue and benevolence. A sensitive and humane surgeon, who can feel sincerely for the sufferers, not only in the contemplation of their cases, but when under his hands during an operation, is sure to inflict little needless pain with them; and as certainly will perform his duty faithfully, because he acts conscientiously. He will never advise or undertake a needless operation; nor will he countenance or excuse such operations in the hands of other surgeons. Every surgical operation is to be regarded, in a degree, as an example of human immolation, offered at the altar of health and life; and it should be approached, accordingly, with feelings of honest sincerity on the part of the surgeon. That is, he must believe, conscientiously, that no other measure will arrest the disease; and that by timely resort to it, health and life, in all probability, may be preserved. These are the cardinal points of operative surgery, from which no surgeon can vary, without compromising his character for honesty, and degrading his noble profession—a humane and benevolent institution, established for the cure of human diseases—to the level of a contemptible stockjobbing occupation, whose objects are, speculating in human blood and human suffering for pecuniary gain or eclat.

A surgeon is not even justifiable in the undertaking of an operation to gratify patients or their friends, when he believes no benefit to the sufferer can possibly result from it. Such an operation would be cruel in the extreme, because its object would be the gratification of hopes founded in the grossest ignorance of the case for which it might be performed, at the expense of great needless bodily suffering to a fellow being.

The most cruel and heartless operations, however, that a surgeon executes, are those designed chiefly for eclat or self-laudation; and there is too much reason to believe that many, nay very many of the operations of the present times are of this description. It is by no means uncommon "now-a-days" to hear of amputation of the limbs, especially the lower, for ulcers, not involving the bones, nor possessing very questionable or menacing characters.

Some few years since, the writer, during an excursion into a neighboring county, chanced to meet with a servant who had lost a leg; and upon interrogating him as to the cause, he replied that it was cut off on account of an ulcer on the skin, not larger than three dollars, that had existed some two or three years, and was caused originally by a burn. The man seemed to be about one or two and twenty years of age; and represented his general health, at the time of the operation, to have been very good. He also informed the writer that the ulcer was not painful at the time the limb was amputated, nor had it ever been so after the burn became an ulcer. The case, from the representations of this mutilated son of Africa, seeming to be one contraindicating the sacrifice of the limb so clearly, and consequently exciting the interest of the writer to know more about it, as well as his sympathy, the man was interrogated more fully, especially as to the advisers and operators in the case; to which interrogatories he readily responded, remarking at the same time that he believed that his leg was cut off just to let the students see the operation, and to bring the doctor, as well as the medical college with which he was connected, into notice.

If the representations of this unfortunate man of his case prior to amputation, were correct, the amputation was not only not demanded, but absolutely improper. And if the operation was resorted to for the purposes surmised by the poor sufferer, it places the surgeon before his enlightened and conscientious brethren as a heartless monster. Amputations of the extremities, particularly of the lower, are seldom required in cases of ulcer; and the writer, who claims to have had much experience with these affections, has never yet found it necessary to resort to amputation for their relief in more than one case out of hundreds of instances, and many of them were unpromising and of long standing.

Even in compound fractures, the sacrifice of limbs will seldom be demanded as indispensable to the preservation of life—as in ulcers of the extremities, the writer's experience has been quite extensive, in treating compound fractures of every grade of violence, and as yet, after more than twenty-five years' intercourse with them, he has not found it necessary to amputate in a solitary case for their relief.

The sacrifice of a limb is indeed an irreparable loss, and should not be resorted to, unless the compound fracture clearly and unequivocally has virtually severed the member from the body, by destroying the chief arteries that naturally supply it with blood. If one principal artery remains intact, in a large majority of cases the limb can be saved by judicious treatment. But when both or most of the principal trunks are entire, even if the contiguous soft parts have suffered extensively, the chances are enhanced for the preservation of the limb nearly in proportion to the integrity of those vessels, and amputation consequently would be improper.

It is in the treatment of these terrific injuries that modern surgery has achieved some of its most brilliant and important cures; and if surgeons would feel less dread of encountering the troubles and disagreeables incident to the treatment of these injuries, and attach less value to Palmer's patents as substitutes for natural limbs, and place the proper estimate upon those supplied by the creative hand, still farther improvements in this important department of surgery would be made, and fewer limbs lost.

The preceding remarks have been suggested from hearing of several surgical operations that had been performed during the past year most wantonly, in cases utterly hopeless, as well as in others not requiring but actually forbidding such operations, as being needless. They were called forth chiefly, however, by some most cruel amputations performed for the cure of rather intractable but no doubt curable ulcers of the leg, involving the soft parts of the limb only. A limb should never be sacrificed because it has an ulcer on it, unless life would be seriously menaced by a longer continuance of the ulcer, or the ulcer possesses malignant characters; and a surgeon who would thus mutilate needlessly should be mulcted with heavy damages for malpractice.—*Virginia Med. Journal.*

